# An Uncommon Cause of Right Hypochondriac Pain

**DOI:** 10.4103/1319-3767.45071

**Published:** 2009-01

**Authors:** Kumble S. Madhusudhan, Shivanand Gamanagatti

**Affiliations:** Department of Radiodiagnosis, All India, Institute of Medical Sciences, New Delhi -110 029, India

A 22-year-old male patient presented to our emergency department with two days' history of upper abdominal pain and fever. There was no history of jaundice. Clinical examination revealed tenderness in the right hypochondrium with mild guarding. There was no palpable mass. Ultrasonography of the abdomen in transverse and longitudinal planes and of one of the small bowel loops is shown in Figure 1. The biliary tree was normal.

**Figure 1:**
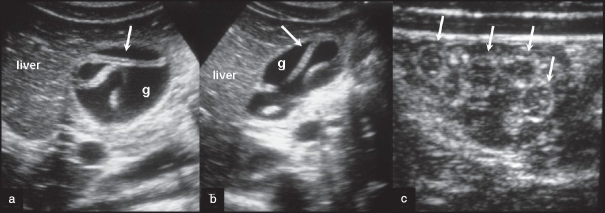
Ultrasonography of the right hypochondrium in transverse (a) and longitudinal (b) planes showing the gall bladder (g). Sonogram of one of the small bowel loops (c) shown with linear transducer

## WHAT IS THE DIAGNOSIS?

## ANSWER

Acute acalculous cholecystitis due to Ascaris lumbricoides with intestinal ascariasis was diagnosed. The patient was managed conservatively, and a weekly dose of albendazole (400 mg) was given for three weeks, and then the patient was lost to follow-up.

Ascariasis is a common parasitic infestation in developing countries. Although small intestine is the primary site, it can be seen in the gall bladder and may cause cholecystitis.[1] Acute cholecystitis develops when there is occlusion of the cystic duct by the worm.[1] Sonography plays an important role in the diagnosis; visualization of a tubular echogenic structure within the gall bladder or bile duct without posterior acoustic shadowing is diagnostic.[2,3] Management is usually medical, with albendazole. Surgery is required in resistant cases.
